# Determinants of delayed onset of lactogenesis II among women who delivered via Cesarean section at a tertiary hospital in China: a prospective cohort study

**DOI:** 10.1186/s13006-022-00523-3

**Published:** 2022-11-30

**Authors:** Weining Lian, Juan Ding, Tiantian Xiong, Jiandi Liuding, LinTao Nie

**Affiliations:** 1 Department of Quality Control, The First Affiliated Hospital of Zhengzhou University, Jianshe Road, Er-Qi District, Zhengzhou City, 450052 Henan Province China; 2 Medical College of Zhengzhou University, 40 Daxue Road, Er-Qi District, Zhengzhou City, 450052 Henan Province China; 3 School of Traditional Chinese Medicine of Nanjing University, 282 Hanzhong Road, Nanjing City, 210029 Jiangsu Province China

**Keywords:** Delayed onset of lactogenesis II, DOLII, Breastfeeding, C section, China

## Abstract

**Background:**

Cesarean birth is associated with a higher prevalence of delayed onset of lactogenesis II (DOLII) than vaginal birth. DOLII refers to the delayed initiation of copious milk production beyond 72 h after birth. This study aimed to determine the prevalence of, and factors associated with, DOLII among women who delivered via Cesarean section in China.

**Methods:**

This prospective longitudinal cohort study recruited 468 women who delivered via Cesarean section at a tertiary hospital in China from 9 October 2021 to 17 May 2022. Face-to-face interviews were conducted during their delivery hospital stay to obtain information about demographic, medical, and breastfeeding factors. We assessed the onset of lactogenesis on postpartum day four, based on the maternal perception of changes in breast fullness. The Edinburgh Postnatal Depression Scale (EPDS) was used to screen for postpartum depression. Women with DOLII were interviewed via telephone or WeChat daily for one week postpartum to determine the timing of the onset of lactogenesis II. Univariate and multivariable logistic regression analyses were used to identify the determinants of DOLII.

**Results:**

DOLII was experienced by 156 of 468 participants (33.3%). After adjusting for potential confounders, the odds of DOLII were 95% higher in primiparous women than multiparous women (adjusted odds ratio [aOR] 1.95; 95% confidence interval [CI] 1.29, 2.98), 75% higher in women with a serum albumin concentration < 35 g / L than women with normal serum albumin concentrations (aOR 1.78; 95% CI 1.09, 2.99), increased by 2.03-fold in women with an EPDS score ≥ 10 than women with an EPDS score < 10 (aOR 2.03; 95% CI 1.35, 3.07), and decreased in women with a higher number of breastfeeding sessions in the first 48 h postpartum (aOR 0.88; 95% CI 0.83, 0.93).

**Conclusions:**

One-third of women with Cesarean section delivery experienced DOLII. DOLII was more likely in women who were primiparous, had a serum albumin concentration < 35 g / L, had a lower frequency of breastfeeding sessions, and had an EPDS score ≥ 10. Women with these risk factors who deliver via Cesarean section may need early breastfeeding support to ensure successful lactation.

## Background

The onset of milk secretion is called lactogenesis, a process that is divided into two stages: secretory differentiation (lactogenesis I) and secretory activation (lactogenesis II) [1]. Lactogenesis I starts at 16 weeks’ gestation. In lactogenesis I, the epithelial cells of the mammary acini differentiate into secretory cells that produce milk, and the mammary gland secretes small amounts of colostrum rich in immunoglobulins. After complete placental removal, progesterone levels decrease sharply and relieve the inhibitory effect of prolactin, triggering the copious milk production that marks the onset of lactogenesis II [2, 3]. Lactogenesis II usually begins between 48 and 72 h postpartum and is felt as breast fullness or engorgement by most women [4, 5]. At the onset of lactogenesis II, mothers perceive an abundant milk flow and are less likely to supplement with formula feeding. However, the onset of lactogenesis II is closely related to the changes in the physiological structure of the mammary epithelial cells, which can be interrupted or delayed by internal and external factors, such as premature delivery or surgical delivery. If the onset of lactogenesis II occurs ≥ 72 h postpartum it is defined as delayed [4, 6]. Delayed onset of lactogenesis II (DOLII) is relatively common, with a prevalence of 12 – 55% among all mothers [5–7]. Previous studies have found that infants of mothers with DOLII experience excessive weight loss (> 10% of birthweight) and therefore often receive formula supplementation [8, 9]. Furthermore, women who experience DOLII are at high risk of premature discontinuation of lactation and shorter duration of breastfeeding [10, 11].

Cesarean delivery is an established risk factor for DOLII [4, 12, 13] and is strongly linked with poorer breastfeeding practices [14–16]. Women giving birth by Cesarean section often have difficulty achieving lactation success owing to postoperative issues such as incision pain and postural limitations; therefore, these women are less likely to initiate breastfeeding [13, 17] and more likely to delay the initiation of breastfeeding [11]. In addition, women who deliver by Cesarean section often have a complex interaction of sociodemographic and maternal–fetal health factors that may influence the onset of lactogenesis II, such as an emergency delivery, macrosomic infant, pregnancy-related complications, preterm birth, and maternal-infant separation.

China has one of the highest prevalences of Cesarean section in the world, reaching 60% in some areas of China [18]. Given the high prevalence of Cesarean birth in China and the association between Cesarean delivery and lactation difficulties, it is critical to identify the risk factors for DOLII so that women with these risk factors receive appropriate care during pregnancy and parturition and are provided with additional support postpartum. To the best of our knowledge, no study has evaluated the prevalence of DOLII after Cesarean section delivery in China. Therefore, we aimed to determine the prevalence of, and factors associated with, DOLII in a cohort of women who delivered via Cesarean section at a tertiary hospital in China.

## Methods

### Study design and setting

This prospective cohort study included women who delivered via Cesarean section at a tertiary hospital in Henan, China, from 9 October 2021 to 17 May 2022. At this hospital, mothers who deliver by Cesarean section are normally discharged on postpartum day five.

### Sampling, inclusion and exclusion criteria

The convenience sample method was applied to identify mothers who met the inclusion and exclusion criteria. The inclusion criteria were: (i) age ≥ 20 years and Cesarean delivery of a singleton who survived; (ii) intent to breastfeed and no obvious contraindications to breastfeeding (e.g., hepatitis B and other active infectious disease diseases; HIV, Treponema pallidum infection; having a neonate with galactosemia or Phenylketonuria); (iii) clear consciousness and ability to understand and answer questions independently; and (iv) available for follow-up via telephone or WeChat. Women were excluded from the cohort if they: (i) had a history of breast surgery including excision biopsies, breast enlargement, breast reduction, or any other surgery involving the breast; (ii) were taking medications that may promote or inhibit lactation after delivery; (iii) had any serious perinatal complications (severe pre-eclampsia or pre-eclampsia, or an NYHA (New York Heart Association) Functional Classification of grade 3 or above); or (iv) had an infant with a critical illness such as cardiopulmonary insufficiency.

### Sample size determination

For the analyses presented in this article, we calculated the sample size based on the formula *n* = Z^2^_1_ − _α/2_ × *pq* /*d*^2^. According to a study conducted in the USA in 2010, the prevalence of DOLII among Cesarean section mothers (estimated *p*) was 59.2% [6]. Based on an allowable deviation of 0.05, statistical power of 90%, and two-tailed significance level of 5%, the minimum required sample size was 300 participants. In anticipation of a 15% dropout rate, we aimed to enroll 345 participants. The actual sample size was 468, indicating that our analyses had sufficient statistical power.

### Definition of variables

#### Outcome variable

DOLII was defined as if maternal perception of lactation occurred after 72 h postpartum. Maternal perception of the onset of lactation was assessed by asking women to recall the presence of breast fullness, swelling, and leaking of milk from the breast, and to recall when these signs of lactation II first occurred. This method has been demonstrated to be a valid clinical indicator of lactogenesis II with a sensitivity and specificity of 71.4% and 79.3%, respectively [19].

#### Independent variables

We selected the variables from potential confounders of the onset of lactogenesis identified in prior studies from other countries [4, 6, 20–25]. Demographic, medical, and breastfeeding factors for the mother and infant were included in the current analysis. The demographic factors were maternal age at delivery, nationality, education level, employment status (any job at the time of birth), mean monthly household income per person, and smoking status and alcohol consumption during pregnancy. The maternal medical factors were gravidity, parity, repeat Cesarean section, pre-pregnancy body mass index (BMI), gestational weight gain (GWG), pregnancy complications, pregnancy course attendance, previous insufficient lactation, serum albumin concentration, hemoglobin concentration, infertility treatment (assisted reproductive technology), insulin treatment, antenatal corticosteroid treatment, type of Cesarean section (elective or emergency), pregnancy outcomes (normal or adverse), type of anesthesia (intraspinal or general), blood loss volume during delivery, and intrapartum fluid volume. The infant medical factors were sex, gestational age, birthweight, height, Apgar score, and neonatal intensive care unit (NICU) admission. The breastfeeding factors were the time of the first breastfeeding session, frequency of breastfeeding in the first 48 h postpartum, and use of formula supplementation during the first 72 h postpartum. The “monthly household income per person” was determined by the total monthly income of all members of the household divided by the total family size. Maternal pre-pregnancy BMI was calculated as weight (kg) / height (m^2^) and used to categorize the participants according to the Chinese adult BMI classification as underweight (< 18.5 kg / m^2^), normal weight (18.5–23.9 kg / m^2^), overweight (24.0–27.9 kg / m^2^), and obese (≥ 28.0 kg / m^2^) [26, 27]. GWG was calculated as the difference between the pre pregnancy weight and the last weight measurement during pregnancy. GWG was categorized according to the Society of Chinese Nutrition recommendations [26], which state that the target GWG should be 11.0–16.0 kg for underweight women, 8.0–14.0 kg for those with normal weight, 7.0–11.0 kg for overweight women, and 5.0–9.0 kg for those with obesity. Maternal GWG was then categorized as excessive, adequate, and inadequate. Pregnancy complications of interest included hypertensive disorders of pregnancy, diabetes (including type 1, type 2, and gestational); thyroid disease (including hypothyroidism and hyperthyroidism); and intrahepatic cholestasis. The maternal serum albumin concentrations (g / L) and hemoglobin concentrations (g / L) were obtained from the medical records of the regular antenatal examinations. Based on our experience, the local normal maternal serum albumin concentration during pregnancy ranged from 35 to 55 g / L. Hemoglobin concentrations were adjusted for altitude as recommended by the World Health Organization [28] to define anemia in pregnant women as a hemoglobin concentration of less than 110 g / L. The hemoglobin concentration of the pregnant women was not adjusted based on their smoking habits because smoking is currently very rare in Chinese women. The prevalence of smoking was 0.2% in the studied population.

### Edinburgh Postnatal Depression Scale [29]

The Edinburgh Postnatal Depression Scale (EPDS) is a previously validated tool to measure postpartum depressive symptoms [30, 31]. The EPDS consists of 10 domains, covering mood, pleasure, self-blame, anxiety, fear, insomnia, coping skills, sadness, crying, and self-harm. The 10 domains are scored by mothers in accordance with the severity of their symptoms as 0 (never), 1 (occasionally), 2 (often), and 3 (always). Hence, the total EPDS score ranges from 0 to 30, with a higher score indicating a higher risk of postpartum depression. As recommended [32], we used a cut-off EPDS score of 10 to indicate that the mother had postpartum depressive symptoms and was at high risk of postpartum depression (EPDS score ≥ 10).

### Data collection

During the seven-month recruitment period, researchers attempted to contact all women who delivered a live infant via Cesarean section within the first 24 h after the birth. Baseline demographic information was obtained via face-to-face interviews conducted by the main author and a trained research assistant. Starting 24 h after delivery, we asked the mothers if their milk had come in, if they had noticed breast fullness, swelling, or leakage, and recalled when these signs of lactation II first occurred. The timing of the onset of lactogenesis II was recorded to the nearest hour. If the mother had not experienced lactogenesis II within the first 72 h postpartum, the woman was in contact via telephone or WeChat (Tencent Holdings Limited, Shenzhen, China) daily and was followed up to seven days postpartum. Data were collected on breastfeeding practices in the first 24 and 48 h postpartum, including the frequency of breastfeeding and formula supplementation. Participants completed the EPDS survey on the day of hospital discharge. Data regarding maternal and infant clinical characteristics and the date and time of the birth were extracted from the medical records.

### Statistical analysis

We examined the associations between the outcome (DOLII) and a set of independent variables to identify the factors associated with DOLII. All data cleaning and preparation were done using Microsoft Excel, version 14.1.0 (Microsoft®, Redmond, WA, USA). We used R statistical software version 4.1.0 (SSRI Company, Ltd., Tokyo, Japan) for all analyses.

We conducted an initial descriptive analysis of the characteristics of the study cohort. All covariates were assessed for normality. Covariates that did not meet the criterion of normality were analyzed using non-parametric methods. Descriptive statistics were presented as mean ± standard deviation for continuous variables, and as median (25th–75th percentile) for non-normally distributed continuous variables. Categorical variables were presented as frequencies and proportions. The chi-squared test (or Fisher’s exact test when the cell size was < 5) was used to detect differences in proportions of categorical variables. The Wilcoxon rank-sum test was used to detect differences in medians of continuous variables.

We applied univariate logistic regression to estimate the unadjusted association between DOLII and potential explanatory factors. Factors showing an independent association with DOLII (*p* < 0.25) were considered eligible for inclusion in the initial multivariable logistic regression model. The cut-off *P*-value was set at a value larger than the level of significance to obtain as many important variables as possible for inclusion in the model. All independent variables in the initial model were examined for collinearity by calculating the variance inflation factor; using a variance inflation factor of ≥ 4 as the threshold for collinearity [33], there was no evidence of collinearity. Following the construction of the initial models, a backward elimination method was used to remove variables that were not significantly associated with DOLII (*p* > 0.05). All variables significant at the α = 0.05 significance level were retained in the final model. We presented the results as unadjusted odds ratios and adjusted odds ratios (aORs), with 95% confidence intervals (CI). The level of statistical significance was set at *p* < 0.05.

### Ethical considerations

This study was approved by the Scientific Research and Clinical Trial Ethics Committee of the First Affiliated Hospital of Zhengzhou University (project identification code: 2022-KY-0104). During the initial interview, the researchers informed the mothers that their participation was voluntary and that they could withdraw at any time without declaring any reason. Written informed consent was obtained from each participant before study participation.

## Results

### Participant flowchart and clinical characteristics

**Fig. 1:**
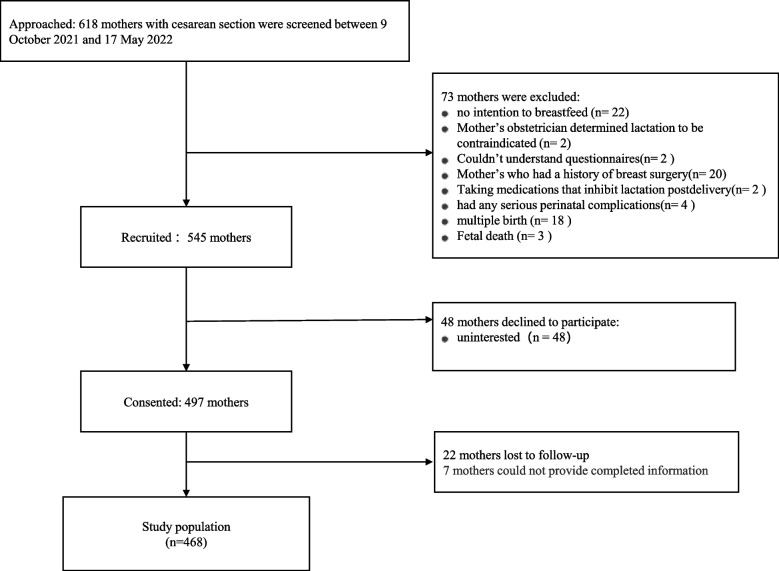
Flowchart of the inclusion and exclusion of study participants

A flowchart of the recruitment and follow-up process is shown in Fig. 1. A total of 618 mothers delivered via Cesarean section between 9 October 2021 and 17 May 2022 and were screened for study eligibility. Of the 618 mothers, 73 were excluded for the following reasons: no intention to breastfeed (*n* = 22); cessation of breastfeeding due to illness (*n* = 2); inability to understand the questionnaires (*n* = 2); history of breast surgery (*n* = 20); intake of medications that inhibit lactation postpartum (*n* = 2); and serious perinatal complications comprising severe preeclampsia (*n* = 3); cardiac function level 3 (*n* = 1); multiple births (*n* = 18) and fetal death (*n* = 3). A total of 545 eligible mothers were initially enrolled in this study. During the study, 48 mothers declined to participate. Although no further questions were asked of those who withdrew, the main reasons cited for withdrawal were maternal exhaustion and lack of interest. At seven days postpartum, another 29 participants were excluded, of whom 22 were lost to follow-up and seven could not provide completed information. Therefore, our analysis included 468 participants.

Table 1 shows the demographic and clinical characteristics of the participants. Most participants were of Han nationality (98.9%), and the remaining were of ethnic minorities (1.1%). The median age of the 468 mothers was 31.5 (29.0–34.0) years. More than half of the women had attained more than 16 years of education, and 265 were employed during their pregnancy. More than 60% of families had a mean monthly household income per person of more than 5,000 remimbi (RMB). Of the 468 mothers, 53.6% (251 / 468) were primiparous; of those who were multiparous, 82.7% (178 / 217) had repeat Cesarean sections. Regarding pregnancy complications, 15.4% (72 / 468) had hypertensive disorders of pregnancy, 30.6% (143 / 468) had gestational diabetes mellitus, and 14.7% (69 / 468) had thyroid disease. Because only 7.0% (10 / 143) of the women with diabetes were treated with insulin, this factor was excluded from further analysis. Among the neonates, 246 were male and 30.1% (141 / 468) were premature; 19.7% (92 / 468) weighed less than 2500 g at birth and 39.1% (183 / 468) were transferred to the NICU after birth. Because most infants (98.1%, 459 / 468) had an Apgar score at one minute of 7–10, this factor was excluded from further analysis. The median time of the initiation of breastfeeding was 10 (4–27) hours; the median number of breastfeeding sessions during 0–24 h and 24–48 h postpartum were 2 (0–4) and 4 (2–8), respectively. More than 98% of infants were fed formula within the first 72 h postpartum; thus, this factor was not considered as a confounder.

**Table 1:** Characteristics of the participants (*n* = 468)

Variable	*n* (%) or median [IQR]
**Maternal characteristics**	
Maternal age (years)	31.5 (29.0, 34.0)
Han ethnicity	463 (98.9%)
Educational level (years)	
≤ 9	62 (13.2%)
10 – 15	147 (31.4%)
≥ 16	259 (55.3%)
Employed	265 (56.6%)
Mean monthly household income per person (RMB)	
< 3000	46 (9.8%)
3001 – 5000	106 (22.6%)
5001 – 10,000	189189189189189118989(40.4%)
> 10000	127 (27.1%)
Smoking status during pregnancy	1 (0.2%)
Alcohol consumption status during pregnancy	8 (1.7%)
First pregnancy	146 (31.2%)
Primiparity	246 (52.6%)
Repeated Cesarean section^a^	182 (82.0%)
Pre-pregnancy BMI (kg/m^2^)	
< 18.5	38 (8.1%)
18.5 – 23.9	265 (56.6%)
24.0 – 27.9	114 (24.4%)
≥ 28.0	51 (10.9%)
GWG^b^	
Adequate	167 (35.7%)
Inadequate	40 (8.5%)
Excessive	261 (55.8%)
Hypertension	72 (15.4%)
Diabetes	143 (30.6%)
Thyroid disease	69 (14.7%)
Ovarian theca-lutein cyst	10 (2.1%)
Pregnancy course attendance	221 (47.2%)
Previous insufficient lactation^b^	54 (24.9%)
Serum albumin (< 35 g / L)	351 (75.0%)
Hemoglobin (< 110 g / L)	140 (29.9%)
Infertility treatment	72 (15.4%)
Insulin GDM treatment^c^	10 (7.0%)
Antenatal corticosteroid treatment	82 (17.5%)
EPDS Score (≥ 10)	216 (46.2%)
**Labor and delivery characteristics**	
Emergency Cesarean	194 (41.5%)
Adverse pregnancy outcomes^d^	160 (34.2%)
Anesthesia type	
Intraspinal	361 (77.1%)
General	107 (22.9%)
Peri-operative bleeding (mL), median [IQR]	200 (200,300)
Intra-operative fluid dosage (mL), median [IQR]	1000 (800,1300)
**Infant and breastfeeding characteristics**	
Male	246 (52.6%)
Gestational age (< 37 weeks)	141 (30.1%)
Birthweight (< 2500 g)	92 (19.7%)
Height, cm	50.0 (47.0, 51.0)
1-min Apgar score (< 7)	9 (1.9%)
NICU admission	183 (39.1%)
Initiation of breastfeeding (hour), median [IQR]	10 (4,27)
Breastfeeding during 0–24 h (times), median [IQR]	2 (0,4)
Formula supplementation during the first 0–24 h	464 (99.1%)
Breastfeeding during 24–48 h (times), median [IQR]	4 (2, 8)
Formula supplementation during the first 24–72 h	461 (98.5%)

Data are presented as number (%) or median (IQR)

*Abbreviations*: *BMI* Body mass index, *EPDS* Edinburgh Postnatal Depression Scale, *GWG* Gestational weight gain, *IQR* Interquartile range, *RMB* Renminbi

^a^ Frequencies and proportions are based on the number of multiparous mothers. There were 222 multiparous women in this study, including 59 in the delayed onset of lactogenesis II group and 163 in the normal onset of lactogenesis II group

^b^ Categorized according to the GWG recommended by Chinese nutritional guidelines: inadequate GWG = weight gain of < 11.0, < 13.0, < 8.0, < 7.0, and < 5.0 kg for underweight, normal weight, overweight, and obese women, respectively; excessive GWG = weight gain of > 16.0, > 14.0, > 11.0, and > 9.0 kg for underweight, normal weight, overweight, and obese women, respectively; adequate GWG = weight gain between the cut-off values for inadequate and excessive GWG

^c^ Frequencies and proportions are based on the number of women with diabetes. There were 143 women with diabetes in the study

^d^ Adverse pregnancy outcomes include preterm birth (< 37 weeks), macrosomia (> 4000 g), low birthweight (< 2500 g), very low birthweight (< 1500 g), and extremely low birthweight (< 1000 g)

### Prevalence of self-reported DOLII and characteristics of women reporting DOLII

Table 2 shows the prevalence of delayed onset of lactogenesis II based on the selected explanatory variables. Of the 468 mothers, 33.3% (156 / 468) failed to achieve lactogenesis II within the first 72 h postpartum. We found evidence of important differences in the proportion of reported DOLII by gravidity. About 41.8% of first-time pregnant women reported DOLII, compared with 29.5% of women with two or more previous pregnancies. Close to 40.0% of the women who were primiparous experienced DOLII, while 26.6% of the multiparous women had DOLII. We also observed that 42.1% among women with EPDS score ≥ 10 had reported DOLII, compared to 25.8% among women with EPDS score < 10. In addition, reporting of DOLII was higher among women who gave birth to newborns with a birthweight less than 2500 g (43.5%) compared to 30.9% among women who gave birth to newborns with a birthweight of more than 2500 g. The proportion of mothers with DOLII whose newborns was transferred to NICU (39.9%) was higher compared to mothers who roomed-in with their newborns (29.1%). Continuous variables and their association with DOLII are shown in Table 3.

**Table 2:** Reporting of delayed onset of lactogenesis II (DOLII) by women according to selected explanatory factors, pooled sample (*N* = 156 women with self-reported delayed onset of lactogenesis II)

	**Frequency of women reporting DOLII**	**Percentage of women reporting DOLII**	***P-*value**
**Maternal characteristics**			
Educational levels (years)			
≤ 9	25	40.3	0.45
10 – 15	48	32.7	
≥ 16	83	32.0	
Employment status			
Employed	87	32.8	0.79
Unemployed	69	34.0	
Mean monthly household income per person (RMB)			
< 3000	13	28.3	0.42
3001 – 5000	42	39.6	
5001 – 10000	62	32.8	
> 10000	39	30.7	
Gravidity (times)			
1	61	41.8	0.012
≥ 2	95	29.5	
Parity			
Multiparous	59	26.6	0.003
Primiparous	97	39.4	
Repeat Cesarean section^a^			
Yes	49	26.9	0.80
No	10	25	
Pre-pregnancy BMI (kg / m^2^)			0.08
< 18.5	8	21.1	
18.5 – 23.9	82	30.9	
24.0 – 27.9	47	41.2	
≥ 28	19	37.3	
GWG^b^			0.62
Adequate	54	32.3	
Inadequate	11	27.5	
Excessive	91	34.9	
Hypertension			
Yes	31	43.1	0.06
No	125	31.6	
Diabetes			
Yes	46	32.2	0.72
No	110	33.8	
Thyroid disease			
Yes	25	36.2	0.58
No	131	32.8	
Ovarian theca-lutein cyst			
Yes	5	50.0	0.31
No	151	33.0	
Pregnancy course attendance			
Yes	64	29.0	0.06
No	92	37.2	
Previous insufficient lactation^a^			
Yes	16	29.6	0.56
No	43	25.6	
Serum albumin (g / L)			
< 35	127	36.2	0.02
≥ 35	29	24.8	
Hemoglobin (g / L)			
< 110	40	28.6	0.15
≥ 110	116	35.4	
Infertility treatment			
Yes	30	41.7	0.10
No	126	31.8	
Antenatal corticosteroid treatment			
Yes	28 (17.9)	34.1	0.86
No	128	33.2	
EPDS Score			
≥ 10	91	42.1	< 0.001
< 10	65	25.8	
**Labor and delivery characteristics**			
Type of Cesarean section			
Emergency	74	38.1	0.06
Scheduled	82	29.9	
Adverse pregnancy outcomes^c^			
Yes	59	36.9	0.24
No	97	31.5	
Anesthesia type			0.70
Intraspinal	122	33.8	
General	34	31.8	
**Infant and breastfeeding characteristics**			
Infant gender			
Male	77	31.3	0.33
Female	79	35.6	
Gestational age (weeks)			
< 37	53	37.6	0.20
≥ 37	103	31.5	
Birthweight (g)			
< 2500	40	43.5	0.02
≥ 2500	116	30.9	
NICU admission			
Yes	73	39.9	0.02
No	83	29.1	

*Abbreviations*: *BMI* body mass index, *EPDS* Edinburgh Postnatal Depression Scale, *GWG* Gestational weight gain, *NICU* Neonatal intensive care unit, *RMB* Renminbi

^*^P-values are based on the results of the chi-squared test for categorical variables

^a^ Frequencies and proportions are based on the number of multiparous mothers. There were 222 multiparous women in this study, including 59 in the delayed onset of lactogenesis II group and 163 in the normal onset of lactogenesis II group

^b^ Categorized according to the GWG recommended by Chinese nutritional guidelines: inadequate GWG = weight gain of < 11.0, < 13.0, < 8.0, < 7.0, and < 5.0 kg for underweight, normal weight, overweight, and obese women, respectively; excessive GWG = weight gain of > 16.0, > 14.0, > 11.0, and > 9.0 kg for underweight, normal weight, overweight, and obese women, respectively; adequate GWG = weight gain between the cut-off values for inadequate and excessive GWG

^c^ Adverse pregnancy outcomes include preterm birth (< 37 weeks), macrosomia (> 4000 g), low birthweight (< 2500 g), very low birthweight (< 1500 g), and extremely low birthweight (< 1000 g)

**Table 3:** Comparisons of continuous variables of women with and without delayed onset of lactogenesis II (*N* = 468)

	**Delayed lactogenesis II onset** **[median (IQR)]**	**Normal lactogenesis II onset** **[median (IQR)]**	*P-*value*
**Maternal characteristics**			
Age (years)	31.0 (29.0, 35.0)	32.0 (29.0, 34.0)	0.68
Peri-operative bleeding (mL)	250.0 (200.0, 307.5)	200.0 (200.0, 300.0)	0.48
Intra-operative fluid dosage (mL)	1100.0 (800.0, 1300.0)	1000.0 (800.0, 1250.0)	0.23
**Infant and breastfeeding characteristics**			
Height (cm)	49.0 (47.0, 50.3)	50 (48.0, 51.0)	0.10
Initiation of breastfeeding (hour)	19 (5.9, 31.6)	8.3 (3.9, 25.0)	< 0.001
Breastfeeding during 0 – 24 h (times)	1 (0, 3)	3 (0, 5)	< 0.001
Breastfeeding during 24 – 48 h (times)	3 (1, 5)	5 (2, 9)	< 0.001

*Abbreviations*: *IQR* Interquartile range

^* ^*P*-values are based on the results of the Wilcoxon rank-sum test for continuous variables

### Logistic regression analyses of factors associated with self‑reported DOLII

**Fig. 2:**
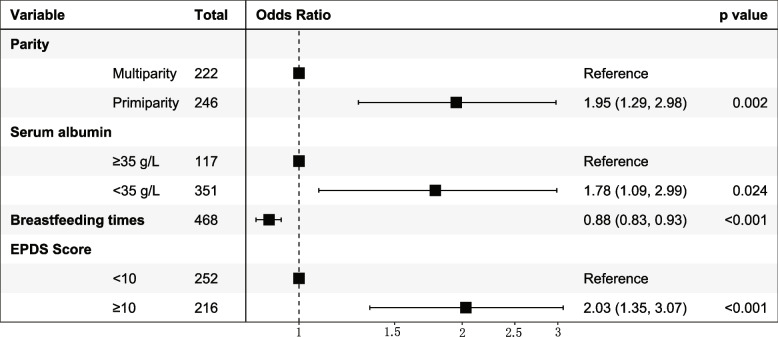
Multivariable predictors of delayed onset of lactogenesis II among Chinese women

Table 4 shows the univariate and multivariable logistic analyses of the associations between the assessed variables and DOLII. Univariate logistic regression analysis to determine the factors associated with DOLII identified 20 variables with *P*-values of < 0.25, which were included in the multivariable logistic regression analysis. At the multivariate level, the factors associated with DOLII were primiparity, serum albumin concentration < 35 g / L, number of breastfeeding sessions, and EPDS score ≥ 10, which were plotted on a forest map drawing (Fig. 2). The multivariable logistic regression results showed that the odds of DOLII were 88% higher for primiparous women than multiparous women (aOR 1.95; 95% CI1.29, 2.98); 75% higher among women with a serum albumin concentration < 35 g / L than those with a normal serum albumin concentration (aOR 1.78; 95% CI 1.09, 2.99); and 2.04 times higher among women with an EPDS score ≥ 10 than those with an EPDS score < 10 (aOR 2.03; 95% CI1.35, 3.07). Women with a higher number of breastfeeding sessions in the first 48 h postpartum had decreased odds of DOLII (aOR 0.88; 95% CI 0.83, 0.93).

**Table 4:** Unadjusted and adjusted odds ratios of factors associated with delayed onset of lactogenesis II

Variables	Unadjusted odds ratio (95% CI)	*P*-value	Adjusted odds ratio (95% CI)^a^	*P*-value
**Maternal characteristics**				
Age (years)	0.99 (0.95, 1.04)	0.86		
Educational levels (years)				
≤ 9	1			
10 – 15	0.72 (0.39, 1.33)	0.289		
≥ 16	0.70 (0.40,1.24)	0.217		
Employed	0.95 (0.64, 1.40)	0.792		
Mean monthly household income per person (RMB)				
< 3000	1			
3001 – 5000	1.67 (0.80, 3.62)	0.183		
5001 – 10000	1.24 (0.62, 2.59)	0.554		
> 10,000	1.13 (0.54, 2.43)	0.756		
First pregnancy	1.71 (1.14, 2.58)	0.009		
Primiparity	1.70 (1.22, 2.67)	0.003	1.95 (1.29, 2.98)	0.002
Repeat Cesarean section	1.11 (0.52, 2.53)	0.803		
Pre-pregnancy BMI (kg / m^2^)				
18.5 – 23.9	1			
< 18.5	0.60 (0.25, 1.30)	0.216		
24.0 – 27.9	1.57 (1.00, 2.47)	0.053		
≥ 28.0	1.33 (0.70, 2.46)	0.377		
GWG				
Adequate	1			
Inadequate	0.79 (0.36, 1.67)	0.555		
Excessive	1.12 (0.74, 1.70)	0.590		
Hypertension	1.64 (0.98, 2.73)	0.059		
Diabetes	0.93 (0.61, 1.40)	0.723		
Thyroid disease	1.16 (0.67, 1.97)	0.580		
Ovarian theca-lutein cyst	2.03 (0.56, 7.41)	0.268		
Pregnancy course attendance	0.69 (0.46, 1.01)	0.058		
Previous insufficient lactation	1.22 (0.61, 2.39)	0.56		
Serum albumin (< 35 g / L)	1.72 (1.08, 2.80)	0.024	1.78 (1.09, 2.99)	0.024
Hemoglobin (< 110 g / L)	0.73 (0.47, 1.12)	0.154		
Infertility treatment	1.53 (0.91, 2.55)	0.105		
Antenatal corticosteroid treatment	1.05 (0.63, 1.72)	0.863		
**Labor and delivery characteristics**				
Emergency Cesarean	1.44 (0.98, 2.13)	0.063		
Adverse pregnancy outcome	1.27 (0.85, 1.90)	0.242		
Anesthesia type				
Intraspinal	1	0.697		
General	0.91 (0.57, 1.44)			
Peri-operative bleeding (ml)	1.00 (0.99, 1.00)	0.342		
Intra-operative fluid dosage (mL)	1.00 (0.99, 1.00)	0.264		
**Infant and breastfeeding characteristics**				
Male	0.82 (0.56, 1.21)	0.326		
Gestational age (< 37 weeks)	1.31 (0.86, 1.98)	0.20		
Height	0.95 (0.90, 0.10)	0.04		
Birthweight (< 2500 g)	1.72 (1.08, 2.75)	0.022		
NICU admission	1.62 (1.09, 2.39)	0.016		
Initiation of breastfeeding (hour)	1.02 (1.01, 1.03)	< 0.001		
Breastfeeding during 0 – 24 h (times)	0.85 (0.78, 0.92)	< 0.001		
Breastfeeding during 24 – 48 h (times)	0.87 (0.83, 0.92)	< 0.001	0.88 (0.83,0.93)	< 0.001
EPDS Score (≥ 10)	2.09 (1.42, 3.10)	< 0.001	2.03 (1.35, 3.07)	< 0.001

*Abbreviations*: *BMI* Body mass index, *CI* Confidence interval, *EPDS* Edinburgh Postnatal Depression Scale *GWG* Gestational weight gain, *NICU* Neonatal intensive care unit, *RMB* Renminbi

^**a**^ Adjusted for educational levels, mean monthly household income per person, gravidity, parity, pre-pregnancy BMI, hypertension, pregnancy course attendance, serum albumin concentrations, hemoglobin concentrations, infertility treatment, type of Cesarean section, adverse pregnancy outcomes, gestational age, infant height, birthweight, NICU admission, onset of breastfeeding, frequency of breastfeeding during 0–48 h postpartum, and EPDS Score

## Discussion

This is the first cohort study to investigate the prevalence of DOLII and its associated factors among mothers who delivered by Cesarean section in China. Our analysis showed that more than one-third of participants experienced DOLII. Factors that independently predicted the occurrence of DOLII were primiparity, maternal serum albumin concentration < 35 g / L, lower frequency of breastfeeding within 48 h postpartum, and EPDS score ≥ 10.

In our study, 66.7% of mothers who delivered via Cesarean section achieved lactogenesis II within three days (0–72 h) postpartum, while 33.3% experienced DOLII. The prevalence of DOLII in our study was lower than that reported in previous studies conducted in other settings. For instance, a cohort study of primiparous women in America reported a prevalence of DOLII among Cesarean section mothers of 53.0% [4]. Another small cohort study conducted in Australia found that 48.7% of women experienced DOLII [23]. In contrast, only 7.9% of breastfeeding women in Peru reported experiencing DOLII [34]. The prevalence of DOLII in our study is similar to the prevalence of 35.5% reported in a Brazilian cohort study [12, 35]. This variation in the prevalence of DOLII between countries may be associated with differences in study design, maternal characteristics, types of hospitals, and breastfeeding practices.

We found that primiparity was an independent risk factor for DOLII, which is consistent with the results of previous studies [6, 7, 23, 36–38]. Zuppa et al. reported [39] lower levels of basal serum prolactin in multiparous women and a faster rise in milk volume during the first few days postpartum compared to primiparous women. They hypothesize that this finding is related to an increase in the number of occupied prolactin receptors in the mammary glands of multiparous women. Furthermore, based on the results of animal experiments described in the literature, mammary tissue is not fully developed in the primordial mammary glands [40]. Part of the development of the mammary glands, due to estrogen and progesterone secretions, also occurs during pregnancy [41]. At the end of the lactation period there is a rapid decrease in the number of mammary epithelial cells, but some remain if the dry lactation period is not prolonged [42]. As a result, epigenetic changes occurring during the first lactation cycle hasten the onset of lactation for subsequent cycles. Multiparous women experience a prolactin spike during lactation and an increase in the number of occupied prolactin receptors in the mammary glands, thus increasing the sensitivity and efficacy of lactation and reducing the risk of DOLII [4]. In addition, primiparous women have less breastfeeding experience, which may have caused them to delay the initiation of breastfeeding and assume an incorrect feeding posture, resulting in reduced effective sucking times of newborns and an increase in DOLII [43]. Also, primiparous women experience greater stress during labor and have a greater fear of childbirth than multiparous women, which may be a factor delaying the onset of lactation [44]. The fear of childbirth can aggravate the sense of labor pain, reflexively cause vagus nerve excitement, and increase the blood adrenalin concentration of parturients, which inhibits the normal onset of the lactation mechanism [45]. Because primiparity is not a modifiable factor, primiparous mothers should be targeted for closer postpartum breastfeeding support to assist them establishing successful breastfeeding prior to discharge.

We found that serum albumin concentrations below the lower limit of the normal range in Cesarean section mothers were a major barrier to the onset of lactogenesis II. A possible explanation for this could be that maternal nutritional status is related to the functional status of the mammary glands [46]. It is well known that serum albumin levels, a long-established biological marker of nutritional status in the body, may be a good proxy for maternal nutritional status. Although the associations between maternal nutritional status and early lactation outcomes have rarely been investigated in previous studies, our findings were concordant with one recent prospective cohort study that reported that the serum albumin concentration was significantly lower in women with DOLII (< 35 g / L) than in those without DOLII (35–50 g / L) [47]. Similarly, another study has shown that the nutritional status of the mother has a decisive effect on the maintenance of milk production. The average amount of milk produced by a nursing mother with poor nutritional status is only about half the amount given daily by a nursing mother with better nutritional status [48]. Further research is needed to investigate the potential association between serum albumin levels and DOLII risk and what role maternal nutrition plays in the lactation process.

We found that a lower number of breastfeeding sessions in the first 48 h postpartum was also associated with a higher risk of DOLII, as reported elsewhere. In our study, a lower number of breastfeeding sessions increased the likelihood of DOLII, with every one-unit increase in frequency of breastfeeding associated with an approximately 12% reduction in the risk of DOLII. Our findings are in agreement with a Singaporean study that showed that mothers who initiated six or more breastfeeding sessions per day before postnatal day three achieved lactogenesis II one day earlier than mothers who independently determined their breastfeeding schedule [24]. Previous research has suggested that milk removal during early infant suckling or regular breastmilk expression may elicit physiologic responses, increase the number of prolactin receptors on lactation cells, and prompt the abundant release of prolactin, which stimulates breast milk production, reduces breastfeeding difficulties, and may therefore reduce the risk of DOLII [22]. However, the median number of breastfeeding sessions per day of the women in our study was 4 (2–8), which was far less than the recommended 8–10 times per day. Therefore, it is possible that their onset of lactogenesis II would have been earlier if they had increased the frequency of breastfeeding or pumping sessions. In addition, using formula to feed infants during hospitalization leads to lower breast stimulation, which reduces perceived milk supply. Nearly all the women in our study supplemented their newborns with formula, thus reducing opportunities for breast stimulation and delaying the onset of lactogenesis II.

In our study, postpartum depression remained significantly associated with DOLII after controlling for covariates. Our findings agreed with those from a study conducted in Brazil that showed that mothers with worse postpartum depression symptoms tend to have DOLII [35]. Mothers with worse depression symptoms tend to present with lower prolactin and higher cortisol concentrations, which may affect the lactation reflex, leading to DOLII [30]. The prevalence of DOLII in our study was much higher than that reported in the literature [35, 49], possibly because of the higher proportions of preterm births and maternal-infant separation in our study than in previous studies. Most preterm infants need to be transferred to the NICU immediately after birth and are therefore separated from their mothers. This physical and emotional separation makes the primipar more depressed, which can lead to increased catecholamine and adrenaline in the blood; adrenaline inhibits the release of prolactin and oxytocin from the anterior and posterior pituitary, which inhibits milk secretion and ejection [50]. In addition, an earlier study suggested that mothers with depressive symptoms may experience less confidence in their ability to breastfeed [51]. Therefore, it is recommended that medical personnel and lactation consultants provide individualized interventions to Cesarean mothers with symptoms of postpartum depression to improve breastfeeding initiation and help them to establish milk production.

Several factors that are generally considered to be associated with DOLII (e.g., pre-pregnancy BMI, and gestational diabetes) showed no association with DOLII in our study. Previous studies have identified pre-pregnancy BMI as an important predictor of DOLII [52, 53], which was not the case after adjusting for potential confounders in our study. In contrast, a prospective study conducted in South Florida to evaluate the factors associated with DOLII found that pre-pregnancy obesity was a significant individual factor associated with DOLII [54]; however, 49% (*n* = 109) of women in their study sample had a BMI of ≥ 30 kg / m^2^. Another study conducted in Northern California also reported that a pre-pregnancy BMI of ≥ 30 kg / m^2^ was independently associated with an increased risk of DOLII in women with gestational diabetes [55], but 39% (*n* = 344) of women in their study sample had a BMI of ≥ 30 kg / m^2^. In our study, only 10.9% (*n* = 51) of women had a BMI of ≥ 28 kg / m^2^; this low proportion of women with obesity may have led to a type II statistical error.

In contrast to previous studies, we did not find an association between gestational diabetes and DOLII in our study. This may have been influenced by the nature of our sample — most participants presented with mild or moderate gestational diabetes, 93.0% of whose condition was controlled with diet and oral hypoglycemic agents rather than insulin treatment. Previous studies have reported that insulin treatment might be an independent predictor of DOLII among women with gestational diabetes [55]. Because only 7.0% (*n* = 10) of the participants in our study were receiving insulin treatment, this factor was not included in further analysis.

A strength of our study is the prospective design. This design allowed us to obtain abundant and accurate covariates, which may have avoided confounding bias. We identified the independent predictors of DOLII and provided valuable insights for the early prediction of lactation status, which will help provide better targeted instructions for Cesarean mothers. In addition, we uniformly measured the serum albumin concentrations during pregnancy instead of using the mother's dietary protein intake to explore the association between the maternal protein status and risk of DOLII, which is the first time this accurate and reliable method has been used to assess the association between the maternal protein status and the timing of the onset of lactogenesis II.

Our study also had some limitations. First, the maternal pre-pregnancy weight in the medical records was self-reported by the participants. Because evidence suggests that women tend to underestimate their weight [56], this may have led to an underestimation of pre-pregnancy BMI. Second, although we adjusted for various covariates that were associated with DOLII, other variables such as maternal sleep, postpartum edema, and activities may require investigation. Third, because our study was carried out at a hospital in China with a referral center for high-risk mothers, the results may not be generalizable to other settings. Therefore, our findings may require verification in a larger, more diverse sample.

## Conclusions

Our study assessed the prevalence of DOLII among women who gave birth via Cesarean section and evaluated the factors responsible for DOLII. We found that the timing of lactogenesis II onset was associated with primiparity, maternal serum albumin concentration, frequency of breastfeeding within the first 48 h postpartum, and postpartum depressive symptoms. DOLII was significantly more likely in women who were primiparous; had a serum albumin concentration < 35 g / L; had a lower number of breastfeeding sessions within the first 48 h postpartum; and had an EPDS score ≥ 10. Our results may help to develop a profile of women at risk of DOLII and allow clinicians to target appropriate breastfeeding interventions and provide support and reassurance when DOLII may be expected. By anticipating DOLII, clinicians may be able to support nursing mothers and prevent prompt transition to formula supplementation due to a misperception of insufficient milk production as opposed to DOLII. However, our results require validation in a large population.

## Data Availability

The datasets generated and analyzed in the current study are not publicly available due to patient confidentiality concerns but are available from the corresponding author on reasonable request.

## Abbreviations

aOR: Adjusted odds ratio
BMI: Body mass index
CI: Confidence interval
DOLII: Delayed onset of lactogenesis II
EPDS: Edinburgh Postnatal Depression Scale
GWG: Gestational weight gain
IQR: Interquartile range
NICU: Neonatal intensive care unit
NYHA: Ney York Heart Association
RMB: Renminbi
